# Changes in postural strategy of the lower limb under mechanical knee constraint on an unsteady stance surface

**DOI:** 10.1371/journal.pone.0242790

**Published:** 2020-11-30

**Authors:** Yi-Ying Tsai, Gwo-Ching Chang, Ing-Shiou Hwang

**Affiliations:** 1 Institute of Allied Health Sciences, College of Medicine, National Cheng Kung University, Tainan City, Taiwan; 2 Department of information Engineering, I-Shou University, Kaohsiung City, Taiwan; 3 Department of Physical Therapy, College of Medicine, National Cheng Kung University, Tainan City, Taiwan; University of Rochester, UNITED STATES

## Abstract

Joint constraint could limit the available degrees of freedom in a kinematic chain for maintaining postural stability. This study investigated adaptive changes in postural synergy due to bracing of bilateral knee joints, usually thought to have a trifling impact on upright stance. Twenty-four young adults were requested to maintain balance on a stabilometer plate as steadily as possible while wearing a pair of knee orthoses, either unlocked (the non-constraint (NC) condition) or locked to restrict knee motion (the knee constraint (KC) condition). Knee constraint led to a significant increase in the regularity of the stabilometer angular velocity. More than 95% of the variance properties of the joint angular velocities in the lower limb were explained by the first and second principal components (PC1 and PC2), which represented the ankle strategy and the combined knee and hip strategy, respectively. In addition to the increase trend in PC1 regularity, knee constraint enhanced the mutual information of the stabilometer angular velocity and PC1 (MI_STBV-PC1_) but reduced the mutual information of the stabilometer angular velocity and PC2 (MI_STBV-PC2_). The MI_STBV-PC1_ was also positively correlated to stance steadiness on the stabilometer in the KC condition. In summary, in the knee constraint condition, postural synergy on the stabilometer was reorganized to increase reliance on ankle strategies to maintain equilibrium. In particular, a stable stabilometer stance under knee constraint is associated with a high level of coherent ankle–stabilometer interaction.

## Introduction

A hierarchy of strategies are designed to maintain balance under postural threats. The first line of defense against falls is the ankle strategy in response to a small perturbation. As a postural threat increases, the hip strategy and stepping response are called into play to counter inertial trunk movements. As compared with the ankle and hip joints [1, 2], the knee joint has received much less attention to its role in postural control, probably because physical constraint of the knee joint seems not to impair postural synergy during upright stance [3]. However, a revised conception based on an uncontrolled manifold approach posited that the knee joint is part of a coordination solution to stabilize the center of mass and head position during quiet stance [4, 5]. Tuned by the bi-articular muscles, the knee joint synchronizes with the ankle and hip joints at 4–8 Hz to fulfill the need for fluctuations around static postural equilibrium [6]. With proprioception information from the knee joint [7, 8], postural responses can be initiated to counter postural destabilization due to surface translation and rotation perturbations [9–11]. According to the interpretations, restriction of knee motion from wearing protective knee orthoses, or due to aging [12] and neurological/orthopedic diseases [13–17], affects gait initiation [14] and dynamic balance [15, 16], and knee motion restriction are also associated with disability [17]. However, the compensatory strategy adopted by the rest of the joints in the kinematic chain in response to the knee motion restriction still remains unclear. This missing piece of information is essential for practitioners who choose orthotic and prosthetic devices or set training programs employing knee motion restriction in rehabilitation clinics.

A stabilometer stance is frequently used to train dynamic standing balance in clinics [18–20]. Most previous studies modeled the stabilometer stance as a single inverted pendulum because the ankle strategy is employed to shift the contact point of the stabilometer and floor along with the projecting line of the center of gravity [21, 22]. However, the classical assumption of an ankle mechanism could oversimplify the inter-joint interaction in the stabilometer stance. This argument is supported empirically by the fact that substantial knee flexion absorbs the impact from the perturbation and reduces the tilt of the trunk in response to forward pitch (anterior–posterior) and roll (medial–lateral) tilting perturbations [10, 23]. Furthermore, because no stepping movement is allowed on a stabilometer, the knee joint is likely essential to feet-in-place balance recovery without causing excessive body angular momentum [24]. Therefore, knee immobilization can be expected to alter coordinative strategies in the lower limbs during stabilometer stance. The objective of this study was to investigate the effect of knee immobilization on stabilometer stance. One particular focus was to characterize the reorganization of postural synergy and kinematic factors that contribute to postural stability in a stabilometer stance with immobilized knees. We hypothesized that 1) the compensatory postural synergy would be available to offset constraint-induced postural destabilization, and 2) the functional benefit of the compensatory postural synergy would increase reliance on the ankle joint (or an ankle-engaging strategy) rather than the hip joint to master fluctuation of the stabilometer.

## Methods

### Participants

Twenty-four healthy adults (12 males, 12 females; mean age: 25.2 ± 4.6 years old; height: 166.6 ± 8.3 cm; mass: 62.7 ± 15.7 kg) were recruited from a university campus. No subjects had any reported history of neurologic, cardiovascular, orthopedic, or traumatic disorders. These subjects had also participated in a previous study of stabilometer stance with bilateral ankle constraints [25]. All participants were provided and signed written inform consent prior to the experiment. In accordance with the Declaration of Helsinki, the experimental protocols were approved by the Institutional Review Board of the National Cheng Kung University Hospital (NO. B-ER-105-062).

### Experimental procedures

Postural control was evaluated using a bipedal stance task. The participants, who wore a pair of length- and range-adjustable post-operative knee orthoses, were directed to maintain a steady upright stance, with both arms naturally hanging at their sides, while standing on a stabilometer (a wooden platform (50 cm × 58 cm) with a consistent curved base (radius: 25 cm; height: 25 cm)). Participants were requested to maintain their stance on the stabilometer as steadily as possible for 30 seconds in an experimental trial. In the non-constraint (NC) condition, the joints of the adjustable knee orthoses were unlocked. The participants were allowed to make use of all joint movements in the lower limbs to minimize postural sway. In the knee-constraint (KC) condition, the bilateral knee orthoses were locked to constrain the knees in a straight knee position (knee extension 180 degrees). Only a very small range of voluntary knee movement (less than 1 degree) was possible in the KC condition. The restriction of knee movement was adequate to induce compensatory movements during the stabilometer stance. The duration of each trial was 30 seconds, and four experimental trials were collected for each of the NC and KC conditions for all participants. The order of KC and NC trials was randomized across the subjects.

### Devices and experimental setting

**Fig 1:**
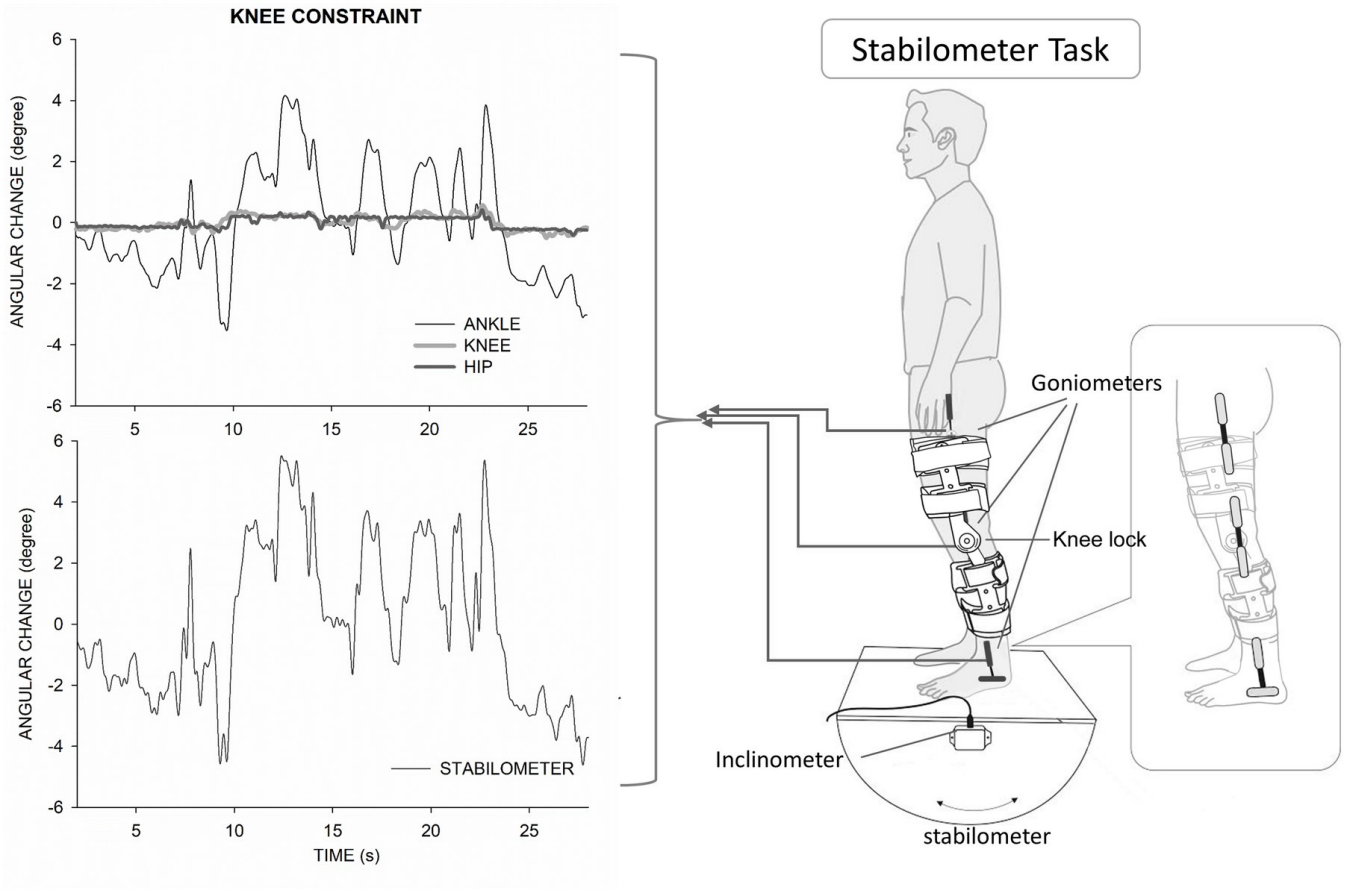
Illustration of the experimental setup and angular excursions in one typical trial in the knee-constraint condition. Illustration of the experimental setup (right plot). Illustration of individual joint angular excursions in one example trial (top left plot). Illustration of stabilometer fluctuation in a typical trial (bottom left plot).

As the angular movements of the dominant limb were sufficient to account for the fluctuating movements of the stabilometer stance [22], the stabilometer tilting angles and angular movements of the dominant ankle, knee and hip joints in degrees were recorded with an inclinometer and electrogoniometers, respectively (Fig 1). The inclinometer (Model FAS-A, LORD MicroStrain, USA) was mounted on the center of the stabilometer to register angular movements of the stabilometer plate. All the electrogoniometers (Model SG110/A and SG150, Biometrics Ltd, UK) were attached to the joint axes of the dominant lower limb, defined as the preferred leg for kicking a ball, to record the angular motions (flexion/extension and dorsiflexion/plantarflexion) in the sagittal plane. For the ankle movement, distal and proximal sensors were placed along the distal lateral aspect of the lower leg just below and above the lateral malleolus, respectively. The distal and proximal sensors for the knee electrogoniometer were attached over the proximal lateral aspects of the leg and the distal lateral thigh, respectively. The axis of the hip joint was the greater trochanter of the femur. The distal arm of the hip electrogoniometer was fastened along the midline of the proximal lateral thigh, and the proximal arm was placed on a projected line between the greater trochanter and the lateral aspect of the iliac crest (Fig 1, right panel). Movement artifacts were minimized by first attaching the electrogoniometers on the skin with double-sided adhesive tape, then wrapping them with surgical tape and binding them with elastic bands. The knee braces were positioned outside the knee electrogoniometer with the joint axis of the brace central to the anatomical knee joint. All participants wore a pair of length and range adjustable post-operative knee orthosis (Nordicare ROM post OP knee brace, Nordicare, Sweden). The length of the knee brace was adjusted to about 2 inches above the lateral malleolus of the ankle and 2 inches below the groin area. All kinematic data were digitized in 1 kHz with an analog-to-digital converter (Model 6341, National Instruments, USA), controlled by a self-written computer program on a LabVIEW platform (LabVIEW v.8.5, National Instruments, USA).

### Data analyses

This study highlighted variations in postural strategy of the lower limb in terms of velocity approach, as velocity information is considered to be the most accurate form for measuring the postural response [26–28]. Angular velocity was derived from the angular displacements registered with the inclinometer and the electrogoniometers of the stabilometer and all the joints. The angular velocities were conditioned with a 4th-order low-pass Butterworth filter (cutoff frequency: 6 Hz). To exclude unstable data at the beginning and the end of an experimental trial, only velocity profiles in the window of interest (2^nd^– 28^th^ second) were analyzed. In the time domain, the size and complexity of the stabilometer velocity profile were represented with root mean square (RMS) and sample entropy (SampEn). The sample entropy was calculated after the velocity profile was down sampled to 100 Hz. The mathematical formula of sample entropy was $SampEn(m,r,N)=-\text{log}(\sum_{i=1}^{N-m}A_{i}/\sum_{i=1}^{N-m}B_{i})$, where *r* = 15% of the standard deviation of the data, *m* is the length of the template (*m* = 3), and *N* is the number of data points in the time series. *A*_*i*_ is the number of matches of the *i*th template of length *m + 1* data points, and *B*_*i*_ is the number of matches of the *i*th template of length *m* data points. Postural sway regularity is an effective biomarker to index attentional investment in postural control [29, 30]. In contrast to randomness, an increase in the regularity (or smaller SampEn) of sway response indicates a higher degree of attentional involvement in postural control. In the spectral domain, the mean frequency (MF) and spectral degree of freedom (DOF) of the stabilometer velocity were determined with the power spectra. The power spectra of stabilometer angular velocity were estimated using a fast Fourier transform and the Welch method (Hanning window, window length: 10 seconds, overlapping time segment: 10% × window length) with a spectral resolution of 0.02 Hz. The MF reflected a spectral shift in stabilometer velocity. Spectral DOF is a statistical approach proposed to estimate spectral dispersion [31]. Spectral DOF is calculated with the following mathematical equation: $\text{DOF}={\left(\sum_{i=1}^{N}S_{i}\right)}^{2}/\sum_{i=1}^{N}S_{i}{}^{2}$, where *S*_*i*_ is the power estimate at each frequency bin in the range of 0–4 Hz. The quantity is unity for a perfectly sharp peak and equal to N (N = 4/0.02 = 200) for white noise.

**Fig 2:**
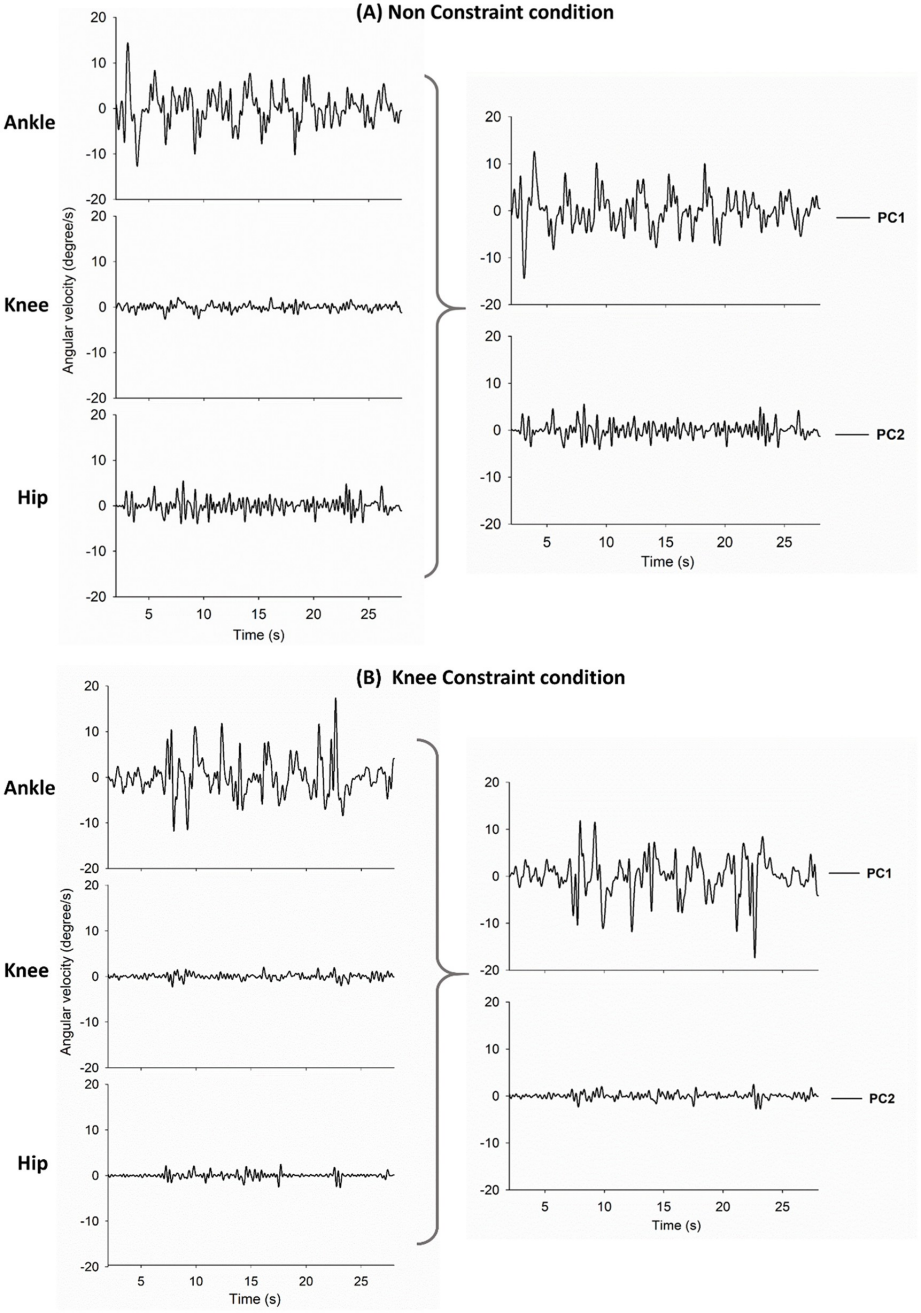
Illustration of a typical trial as an example to demonstrate the NC (A) and KC (B) conditions. Illustration of individual joint angular velocities in one example trial (left plots). Illustration plots of 1^st^ and 2^nd^ principal components (right plots).

Principal component analysis (PCA) was applied to the angular velocities of the ankle, knee, and hip joints to convert those variables into a simpler set of independent components (or PCs). These PCs characterized the multi-segmental postural strategies by reducing the dimensionality of the data set [32, 33]. In effect, PC1 and PC2 were the time series of linear combinations of the angular velocities of the ankle, knee, and hip joints (Fig 2(A) and 2(B)). On average, PC1 could account for more than 80% of the variance properties of the joint angular velocities, and more than 95% of the kinematic variance properties were jointly represented by PC1 and PC2 for both stance conditions. The relative contributions of an individual joint’s velocities to PC1 and PC2 were assessed by squaring the values of the correlation coefficient of the PC1/PC2 and detrended angular velocities of all the joints, or PC1/PC2 communalities (*h*^*2*^_*PC1*_ and *h*^*2*^_*PC2*_). A larger value of communality indicated a greater contribution from the individual joint to this PC. Hence, contrasts of *h*^*2*^_*PC1*_ and *h*^*2*^_*PC2*_ between the NC and KC conditions indicated a constraint effect on each joint movement contributing to postural synergy [25, 34, 35]. After down-sampling to 100 Hz, the size and complexity of the PC1/PC2 were assessed with RMS and SampEn, respectively. Because the viscous resistance of the musculotendon system may attenuate the transmission of high-frequency interactive forces across joints, the couplings of stabilometer velocity (STBV) and PC1/PC2 were assessed with non-linear mutual information (MI_STBV-PC1_ and MI_STBV-PC2_). Mutual information (*MI*(*X; Y*)) (*MI*(*X*; *Y*)) was defined as $MI(X;Y)=\sum_{y\in Y}\sum_{x\in X}p(x,y)\text{log}(p(x,y)/p_{1}(x)p_{2}(y)$), where *p*(*x*, *y*) is the joint probability density function of stabilometer velocity (*X*) and detrended PC1 (or PC2) (*Y*), and p_1_(x) and p_2_(y) are the marginal probability density functions of the two time series, respectively. MI between stabilometer velocity and randomized PC1/PC2, or the lower bound for the STBV-PC coupling, was calculated to examine the significance of MI_STBV-PC1_ and MI_STBV-PC2_. The randomized mean PC1/PC2 was obtained by reshuffling the detrended PC1/PC2 time series. MI_STBV-PC1_ and MI_STBV-PC2_ were significant when MI_STBV-PC1_ and MI_STBV-PC2_ exceeded the lower bound for the STBV-PC coupling.

### Statistical analyses

The primary interest of this study was to investigate the effect of knee constraint on sway dynamics and postural synergy during stabilometer stance. Hotelling’s T- squared test was conducted to compare postural variables in temporal domain (the RMS and SampEn of fluctuations in stabilometer velocity) and the postural variables in frequency domain (the MF and spectral DOF of fluctuations in stabilometer velocity), PC variables (variance property that PC1/PC2 explained, *h*^*2*^_*PC1*_*/h*^*2*^_*PC2*_, RMS, and SampEn of PCs), and stabilometer–PC coupling (MI between stabilometer velocity and PC1/PC2 (MI_STBV-PC1_, MI_STBV-PC2_)) between the NC and KC conditions. Post hoc t test and the effect size (Cohen’s d value) were calculated to identify the specific difference if the main effect of the stabilometer postural variables, PC variables and stabilometer-PC coupling reached significance level (p < 0.05). The Simes test was used to adjust the significant level to prevent inflation of theα value from multiple comparisons [36]. The secondary interest of this study was to examine potential predictors of PC variables for the amount of postural sway for the KC and NC conditions. Pearson’s correlation was used to examine the significance of fluctuations in stabilometer velocity between PC variables (RMS, SampEn, and stabilometer–PC coupling) in the NC and KC conditions. All statistical analyses were computed in the SPSS statistical package 17.0 (SPSS Inc., Armonk, NY, USA), and the significance level was set at 0.05.

## Results

### Changes in stabilometer performance

Table 1 contrasts the kinematic variables of stabilometer angular velocity in the temporal and spectral domains between the NC and KC conditions. The results of Hotelling’s T-squared test revealed a significant difference in temporal variables (RMS and SampEn) (Wilks’Λ = 0.711, *p* = 0.024), rather than in spectral variables (MF and spectral DOF) of stabilometer angular velocity (*p* > 0.05). Post hoc test further indicated that only the SampEn of stabilometer velocity was reduced with knee constraint (*p* = 0.009, *d* = 0.372) due to loss of kinematic complexity with braced knees.

**Table 1:** The contrast of stabilometer angular velocity variables in the temporal (RMS and SampEn) and spectral (MF and spectral DOF) domains between the non-constraint (NC) and knee constraint (KC) conditions.

	Non-Constraint	Knee Constraint	*Statistics*	
			*Hotelling’s T*^*2*^	*Post hoc*
**RMS**	5.626 ± 0.652	5.968 ± 0.514	**Wilks’ Λ = 0.711, *p* = 0.024**	t_23_ = -0.993, *p* = 0.331
**SampEn**	**0.342 ± 0.154**	**0.314 ± 0.158**		**t**_**23**_ **= 2.836, *p* = 0.009, *d =* 0.372**
**MF**	1.448 ± 0.066	1.443 ± 0.065	Wilks’ Λ = 0.969, *p* = 0.706	t_23_ = 0.154, *p* = 0.879
**Spectral DOF**	90.397 ± 3.411	92.243 ± 3.848		t_23_ = -0.723, *p* = 0.477

RMS: root mean square(degree/sec); SampEn: sample entropy; MF: mean frequency (Hz); Spectral DOF: Spectral degree of freedom

### Alternations of the principle components

Table 2(A) shows the percentages of the variance properties that PC1 and PC2 shared in these two stance conditions. PC1, the predominant factor of the coordination solution to stabilometer stance, explained more than 80% of the total variance of joint angular velocities in the NC (82.09 ± 1.94%) and KC (86.81 ± 1.57%) conditions. The results of Hotelling’s T-squared test revealed a significant difference in the variance that PC1, PC2 and PC1+PC2 explained in two stance conditions (Wilks’ Λ = 0.571, *p* = 0.007). Post hoc test further indicated that PC1 explained more variance properties of the joint angular velocities (*p* = 0.001, *d* = 0.547), whereas PC2 accounted for fewer variance properties of the joint angular velocities in the KC condition than in the NC condition (*p* = 0.008, *d* = 0.536). Table 2(B) is the results of Hotelling’s T-squared test to contrast the communalities (*h*^*2*^_*PC1*_ and *h*^*2*^_*PC2*_) of PC1/PC2 with individual joints between the NC and KC conditions. PC1 had the highest communality (*h*^*2*^_*PC1*_) with ankle angular velocity, in support of the critical role of the ankle joint for stabilometer stance in both stance conditions. In contrast, PC2 had relatively higher communality (*h*^*2*^_*PC2*_) with the knee/hip angular velocities than with that of the ankle. This fact indicated that PC2 reflected a combined knee and hip strategy. Also, communality of PC1 (*h*^*2*^_*PC1*_) and PC2 (*h*^*2*^_*PC2*_) with the angular velocities of all the lower limb joints did not vary with knee constraint (*p* > 0.05). Despite the main effect of the structure of PC1 and PC2 with knee constraint being close to but not reaching the statistically significant level (Wilks’ Λ = 0.769, *p* = 0.056), the post hoc test was still conducted to reveal the trend of lower SampEn of the PC1 in the KC condition than in the NC condition (*p* = 0.015, *d* = 0.387). Table 4 contrasts the mutual information (MI) of the angular velocity of the stabilometer (STBV) and PCs between the NC and KC conditions. The group effect of the MI_STBV-PCS_ was significant (Wilks’ Λ = 0.668, *p* = 0.012) and the result of the post hoc test showed that MI_STBV-PC1_ was significantly greater in the KC condition than in the NC condition (*p* = 0.048, *d* = 0.365), whereas MI_STBV-PC2_ was significantly smaller in the KC condition than in the NC condition (*p* = 0.006, *d* = 0.260).

**Table 2:** (A) The amounts of variance that the first two principal components (PC1 and PC2) explain. (B) Communality of PC1 (*h*^*2*^_*PC1*_) and PC2 (*h*^*2*^_*PC2*_) in the two stance conditions.

(A)					
	**Non-Constraint**	**Knee Constraint**		*Statistics*	
				*Hotelling’s T*^*2*^	*Post hoc*
**PC1 (%)**	**82.09 ± 1.94**	**86.81 ± 1.57**		**Wilks’ Λ = 0.571, *p* = 0.007**	**t**_**23**_ **= -3.65, *p* = 0.001, *d* = 0.547**
**PC2 (%)**	**13.99 ± 1.38**	**10.60 ± 1.19**			**t**_**23**_ **= 2.878, *p* = 0.008, *d* = 0.536**
**PC1+PC2 (%)**	**96.08 ± 0.68**	**97.41 ± 0.63**			**t**_**23**_ **= -3.176, *p* = 0.004, *d* = 0.410**
(B)					
		**Non-Constraint**	**Knee Constraint**	*Statistics*	
				*Hotelling’s T*^*2*^	*Post hoc*
***h***^***2***^_***PC1***_	**Ankle**	0.876 ± 0.057	0.920 ± 0.045	Wilks’ Λ = 0.831, *p* = 0.265	t_23_ = -1.712, *p* = 0.100
	**Knee**	0.142± 0.041	0.095 ± 0.019		t_23_ = 1.015, *p* = 0.321
	**Hip**	0.146 ± 0.041	0.155 ± 0.049		t_23_ = -0.214, *p* = 0.832
***h***^***2***^_***PC2***_	**Ankle**	0.091 ± 0.038	0.060 ± 0.032	Wilks’ Λ = 0.746, *p* = 0.098	t_23_ = 1.241, *p* = 0.227
	**Knee**	0.422 ± 0.072	0.267 ± 0.062		t_23_ = 2.507, *p* = 0.020
	**Hip**	0.492 ± 0.064	0.665 ± 0.060		t_23_ = -2.716, *p* = 0.012

### Parameters correlated to better stabilometer performance

The correlations of the PC variables with the RMS of stabilometer angular velocity are summarized in Table 5(A). For both stance conditions, the RMS of stabilometer velocity was positively correlated with the size of PC1/PC2 (*r* = 0.460–0.864, *p* < 0.05). In addition, the RMS of stabilometer velocity was positively correlated with the SampEn of PC1 (*r* = 0.441–0.579, *p* < 0.05) rather than with that of PC2 (*p* > 0.05). This indicated that better stance stability (smaller RMS_STBV_) was associated with a smaller size of PC1/PC2 and higher regularity of PC1. Especially in the KC condition, better stance stability was linked to higher mutual information between the stabilometer and PC1 (MI_STBV-PC1_), as shown in Table 5(B) (*r* = -0.491, *p* = 0.015).

## Discussion

Using principal component analysis, this study demonstrated the reorganization of postural synergies due to bilateral knee bracing. The knee constraint enhanced the regularity of the angular velocity of the stabilometer movement and the amount of kinematic variance represented by PC1, which was mainly contributed by the ankle angular velocity. Knee constraint also resulted in marginally higher regularity of PC1 and stronger coupling between the stabilometer and PC1 in the KC condition. During the knee constraint condition, participants who showed superior kinematic coupling between the stabilometer velocity and PC1 exhibited smaller stabilometer velocities.

### Increase in sway velocity regularity due to bilateral knee constraints

Analogous to that of a quiet stance on a level surface, postural control during stabilometer stance relies mainly on the ankle joints. The ankle joints regulate the contact point of the stabilometer with the floor in response to the projection of the center of gravity [21, 37]. The body oscillates around the axis of the ankle joint like an inverted pendulum [21, 22], as the central nervous system (CNS) co-contracts muscle antagonist pairs crossing the knee and hip joints for frugal use of biomechanical degrees of freedom during stabilometer stance [22]. However, some researchers have challenged this viewpoint from a multi-joint organization perspective [37–39]. For instance, stiffness of the knee joint can vary flexibly with sensory contexts (such as visual and proprioceptive inputs) to achieve stance stability [37]. Although the size of the stabilometer angular velocity was not potentiated, the participants with braced knees became more attentive to postural control [29, 40], as indicated by the regularity enhancement of stabilometer velocity (Table 1). This attentional interpretation of increased sway regularity has also been observed in the elderly [41] and patients with neurological disorders [42, 43], who have greater perceptual load and attentional control for maintaining balance. In this study, bilateral knee constraints, which altered the mechanical states of the kinematic chain in the lower limb, resulted in alternative postural strategies along the anterior–posterior challenge. Hence, the knee joint was not of trifling importance to the stabilometer stance.

### Preferential use of the ankle strategy with knee constraint

Using PCA to represent multi-segment kinematics [25, 44], this study separated posture synergy into two eigen-movements (PC1 and PC2) in the joint space during a stabilometer stance. PC1, with high *h*^*2*^_*PC1*_ with ankle angular velocity, represented the ankle strategy, and PC2, with relatively high *h*^*2*^_*PC2*_ with knee/hip angular velocity, represented the combined knee–hip strategy (Table 2(B)). Knee constraint significantly increased the kinematic variance properties explained by PC1, together with a reduction in the kinematic variance explained by PC2 (Table 2(A)). These results indicated increased reliance on a focal joint (the ankle joint) and minimized the use of non-focal joints (the knee and hip joints) when the knee joint was constrained. In addition, a notable finding was the trend of constraint-induced enhancement in PC1 regularity (smaller SampEn) (Table 3), which corresponded to a decrease in the entropy measure of stabilometer movement (Table 1). The present results indicated an ankle-engaging strategy for greater attentional investment in the ankle joint in the KC condition [29, 40], especially that the SampEn of PC2 was not affected by knee constraint. As compared with the NC condition, knee constraint added more coherent stabilometer–ankle interaction (or higher MI of stabilometer velocity and PC1), with relative decoupling between the stabilometer and PC2 (or lower MI of stabilometer velocity and PC2) (Table 4). A possible functional merit of coupled ankle-stabilometer movement was the reduction of the passive transmission of inertial forces to the kinematic chain above the ankle joint, which thereby effectively realigned the center of gravity to the point of contact of the stabilometer and the floor with the ankle joint [21]. Hence, accessory motions in the non-focal joints (like PC2) could be minimized.

**Table 3:** The contrast of the size and complexity of PC1 and PC2 between the non-constraint and knee constraint conditions.

		Non-Constraint	Knee Constraint	*Statistics*	
				*Hotelling’s T*^*2*^	*Post hoc*
**PC1**	**RMS**	3.229 ± 0.283	3.222 ± 0.205	Wilks’ Λ = 0.769, *p = 0*.*056*	t_23_ = 0.033, *p* = 0.974
	**SampEn**	**0.268 ± 0.015**	**0.244 ± 0.012**		**t**_**23 =**_ **2.321, *p* = 0.015, *d* = 0.387**
**PC2**	**RMS**	1.166 ± 0.119	0.956 ± 0.073	Wilks’ Λ = 0.795, *p* = 0.080	t_23_ = 2.621, *p* = 0.030
	**SampEn**	0.353 ± 0.023	0.356 ± 0.020		t_23 =_ -0.221, *p* = 0.827

RMS: root mean square (degree/sec); SampEn: sample entropy

**Table 4:** The contrast of mutual information (MI) between stabilometer angular velocity (STBV) with PC1 and PC2 between the non-constraint and knee-constraint conditions.

MI	Non-Constraint	Knee Constraint	*Statistics*	
			*Hotelling’s T*^*2*^	*Post hoc*
**STBV-PC1**	**0.167 ± 0.014**	**0.192 ± 0.014**	**Wilks’ Λ = 0.668, *p* = *0*.*012***	**t**_**23**_ **= -2.086, *p* = 0.048, *d* = 0.365**
**STBV-PC2**	**0.142 ± 0.023**	**0.115 ± 0.019**		**t**_**23**_ **= 3.048, *p* = 0.006, *d* = 0.260**

Lower bound for the force-discharge relations: 0.002

Regardless of the stance condition, the degree of postural sway (or stabilometer movement) was positively correlated with the sizes of both PC1/PC2 and SampEn of PC1 (Table 5(A)). Namely, those participants who were more attentive to ankle control in fine-tuning the limb movements could exhibit better stance stability (smaller RMS_STBV_). In the KC condition, a participants who coped with postural destabilization with superior coherent ankle–stabilometer interaction could also maintain better stance stability as well (Table 5(B)).

**Table 5:** Correlation between the amount of stabilometer angular velocity (RMS_STBV_) and key PC variables for the non-constraint and knee constraint conditions. (A). Correlation between RMS_STBV_ and PC size and complexity, (B). Correlation between RMS_STBV_ and PC-STBV coupling (MI_STBV-PCS_).

(A)					
N = 24		**RMS**_**PC1**_	**RMS**_**PC2**_	**SamEn**_**PC1**_	**SamEn**_**PC2**_
**Non Constraint**	**RMS**_**STBV**_	***r* = 0.854, *p* < 0.000**	***r* = 0.699, *p* < 0.000**	***r* = 0.439, *p* = 0.031**	*r* = 0.214, *p* = 0.323
**Knee Constraint**	**RMS**_**STBV**_	***r* = 0.864, *p* < 0.000**	***r* = 0.460, *p* = 0.024**	***r* = 0.579, *p* = 0.003**	*r* = 0.087, *p* = 0.688
(B)					
n = 24		**MI**_**STBV-PC1**_	**MI**_**STBV-PC2**_		
**Non Constraint**	**RMS**_**STBV**_	*r* = -0.200, *p* = 0.349	r = -0.039, *p* = 0.858		
**Knee Constraint**	**RMS**_**STBV**_	***r* = -0.491, *p* = 0.015**	r = -0.228, *p* = 0.284		

MI: mutual information, RMS: root mean square, SampEn: sample entropy

This study got to the essence of the shift in coordination dynamics toward a pure ankle strategy, once the non-focal knee joints were constrained during the stabilometer stance. In our previous study [25], we reported that constraining the focal ankle joint results in shifts in coordination dynamics toward a knee-engaging strategy on the stabilometer. The ankle-knee complex is a strong structural component within the postural synergy framework, supported by rich heteronymous proprioceptive connections [45, 46] and topological overlapping representations in the primary motor cortex between the two adjacent joints [47]. The interchangeable role of the ankle and knee joints during joint constraint could be centrally and/or peripherally pre-programmed.

### Methodological concerns

In this study, we applied PCA to joint angular velocities rather than to joint angular displacements, as was done in the previous work that investigated postural synergy reorganization during ankle constraint [25]. Thanks to the velocity-based approach, changes in postural synergy during the non-focal joint constraints became evident. The findings and claims associated with the velocity-based approach should be further discussed from two perspectives. First, angular velocities obtained by derivatives of angular displacements highlight higher-frequency ingredients within the kinematic data to differentiate variations in the postural synergy between the non-constraint and constraint conditions. In fact, knee movement is often synchronized with the movement of the adjacent joints at higher frequency bands [6, 48], especially for in-phase ankle-knee movements during stabilometer stance [37]. Secondly, it is known that cerebellar function puts emphasis on real-time control of movements. The cerebellum encodes multiple kinematic parameters of movement, including position, velocity, and acceleration, as well as position error [49–51]. In particular, the cerebellar units compute the velocity vectors required for coordinated movements while standing on a slow-moving or unstable surface [52, 53]. In addition, velocity information is coded in neurons of the primate primary motor cortex, which leads to motor behavior preceding position information by about 100 ms [54]. Image studies have indicated the brain areas that are involved in the control of speed during a motor sequence of the foot [55]. Surprising recent findings show that velocity information is the most accurate form deciphered from sensory information for postural stabilization [26–28]. Hence, it was not surprising that velocity dynamics were relevant to highlight the characterization of the subtle but significant the constraint effect impact on postural synergy of the lower limb.

One major limitation of this study was the recruitment of young healthy individuals, instead of disabled subjects with knee motion limitations, whose adaptive strategy to knee constraint might be different from that of healthy adults. Another potential limitation is that both knees were constrained, which promotes symmetry, in comparison to a unilateral knee condition, which affects strength/pain/proprioception. Finally, this study simply specified the postural synergy of the lower limb without considering the accessory movements of the head, trunk and upper limbs. Despite this potential limitation, the findings still provided sufficient insights into adaptive strategies to knee restriction, because posture balance on the seesaw was kept mainly in the hip, knee and ankle joints [22, 37]. For individuals with limited knee range of motion (such as those who wear a long knee brace or locked prosthetic knee), the ankle joint is especially essential for the need for dynamic balance during daily activity.

## Conclusions

Although the knee joint is the secondary joint in maintaining stabilometer stance, constraint of the knee joint requires more attentional investment against postural destabilization in stabilometer stance, as indicated by increases in the regularity of stabilometer velocity. Bilateral knee constraint altered the coordination solution for success in a balanced posture on the stabilometer, with increasing reliance on the ankle strategy. Skilled stabilometer stance with braced knees manifested with a higher degree of velocity coherence between the ankle and stabilometer movement.

## Supporting information

S1 File (XLSX)
